# Prediction of PD-L1 tumor positive score in lung squamous cell carcinoma with H&E staining images and deep learning

**DOI:** 10.3389/frai.2024.1452563

**Published:** 2024-12-20

**Authors:** Qiushi Wang, Xixiang Deng, Pan Huang, Qiang Ma, Lianhua Zhao, Yangyang Feng, Yiying Wang, Yuan Zhao, Yan Chen, Peng Zhong, Peng He, Mingrui Ma, Peng Feng, Hualiang Xiao

**Affiliations:** ^1^Department of Pathology, Daping Hospital, Army Medical University, Chongqing, China; ^2^The Key Lab of Optoelectronic Technology and Systems, Ministry of Education, Chongqing University, Chongqing, China; ^3^Department of Information, Affiliated Tumor Hospital of Xinjiang Medical University, Urumchi, China

**Keywords:** lung squamous cell carcinoma, PD-L1, deep learning, TransUnet, H&E staining

## Abstract

**Background:**

Detecting programmed death ligand 1 (PD-L1) expression based on immunohistochemical (IHC) staining is an important guide for the treatment of lung cancer with immune checkpoint inhibitors. However, this method has problems such as high staining costs, tumor heterogeneity, and subjective differences among pathologists. Therefore, the application of deep learning models to segment and quantitatively predict PD-L1 expression in digital sections of Hematoxylin and eosin (H&E) stained lung squamous cell carcinoma is of great significance.

**Methods:**

We constructed a dataset comprising H&E-stained digital sections of lung squamous cell carcinoma and used a Transformer Unet (TransUnet) deep learning network with an encoder-decoder design to segment PD-L1 negative and positive regions and quantitatively predict the tumor cell positive score (TPS).

**Results:**

The results showed that the dice similarity coefficient (DSC) and intersection overunion (IoU) of deep learning for PD-L1 expression segmentation of H&E-stained digital slides of lung squamous cell carcinoma were 80 and 72%, respectively, which were better than the other seven cutting-edge segmentation models. The root mean square error (RMSE) of quantitative prediction TPS was 26.8, and the intra-group correlation coefficients with the gold standard was 0.92 (95% CI: 0.90–0.93), which was better than the consistency between the results of five pathologists and the gold standard.

**Conclusion:**

The deep learning model is capable of segmenting and quantitatively predicting PD-L1 expression in H&E-stained digital sections of lung squamous cell carcinoma, which has significant implications for the application and guidance of immune checkpoint inhibitor treatments. And the link to the code is https://github.com/Baron-Huang/PD-L1-prediction-via-HE-image.

## 1 Background

Lung cancer is a malignant tumor with significant morbidity and mortality rates. Whereas non-small-cell lung cancer (NSCLC) comprises 80% of all lung cancers, the main types include adenocarcinoma (32–40%), squamous (25–30%), and large cell (8–16%) (Zarogoulidis et al., 2013). Immune checkpoint inhibitors (ICIs) have shown remarkable efficacy in the clinical treatment of NSCLC. Expression of programmed death ligand 1 (PD-L1) is significant for ICI efficacy. Therefore, immunohistochemical staining (IHC) for PD-L1 expression has been approved as a companion diagnostic marker in the clinical treatment of ICIs. However, there are disadvantages to using IHC to detect PD-L1 expression, including high detection and time costs, inconsistent interpretation standards, and the need for strong professional knowledge (Shamai et al., 2022). Furthermore, the interpretation criteria for PD-L1 vary significantly among tumor types. Meanwhile, factors including tumor heterogeneity, the complexity of the immune microenvironment, and the atypical expression of tumor cells presented by IHC staining easily cause subjective errors in the interpretation results of pathologists (Wang J. et al., 2019; Wang S. et al., 2019).

Hematoxylin–eosin (H&E), the most developed staining method in clinical pathology, enables the distinct visualization of cell morphology and tissue structure and is economical and easily operable. With the advancement of technology, digital high-resolution whole slide images (WSI) obtained from H&E-stained slides are used to provide a new direction for artificial intelligence (AI) assisted diagnosis using deep learning methods (Litjens et al., 2016). In recent years, researchers have found that the application of deep learning to pathological H&E-stained images can complete tasks that can be recognized by the human eye, such as tumor classification and tumor grading (Hu et al., 2021; Graham et al., 2019a,b). Moreover, it has a good predictive effect on higher-order tasks, such as gene mutation and survival period prediction (Zeng et al., 2019; Graham et al., 2019a,b) This method can effectively alleviate the problems of traditional PD-L1 detection technology, including its high cost, low efficiency, and subjective interpretation differences.

Wu et al. proposed a high-precision AI system to automatically evaluate the tumor positive cell score (TPS) for NSCLC PD-L1 expression (22C3 and SP263), and the calculated results showed a high degree of agreement between the AI and the pathologist (Wu et al., 2022). Mayer et al. proposed a convolutional neural network (CNN) classification model. Fusions of anaplastic lymphoma kinase (ALK), and ROS proto-oncogene 1 receptor tyrosine kinase (ROS1), were predicted directly from the H&E-stained WSI of postoperative tissues of patients with NSCLC. The sensitivity and specificity of the classifier for ALK and ROS1 were 100 and 98.6%, respectively (Mayer et al., 2022). Shamai et al. developed a WSI classification dataset for H&E staining of breast cancer and predicted the PD-L1 state of H&E-stained images using deep learning technology. The area under the curve (AUC) value of the predicted result was 0.91–0.93 2. (Sha et al., 2019) used deep learning to predict the PD-L1 status of H&E-stained WSIs in NSCLC tissues, with an AUC value of 0.8.

Considering the tissue structural complexity and heterogeneity of lung adenocarcinoma, lung squamous cell carcinoma was chosen as the object in this study. We constructed a WSI dataset using H&E-stained lung squamous cell carcinoma. To further quantitatively predict TPS, a deep learning model was employed for the first time to segment the PD-L1 expression region in H&E-stained WSIs of formalin-fixed and paraffin-embedded (FFPE) lung squamous cell carcinoma samples. The deep learning framework adopted the recently released deep segmentation network TransUnet (Chen et al., 2021), which introduced advanced transformer technology based on the classic Unet model to enhance the model’s ability to acquire contextual structural information in pathological images. This solved the problem wherein traditional convolutional networks exclusively focus on local knowledge, leading to segmented regions with limited structural similarity. Based on H&E-stained WSIs, the model can assist pathologists in predicting PD-L1 TPS and achieving end-to-end prediction.

## 2 Methods

### 2.1 Datasets

We enrolled surgical excision samples of lung squamous cell carcinoma. From January 2018 to December 2021, FFPE tissue samples from lung squamous cell carcinoma with PD-L1 test results from the Department of Pathology, Dapping Hospital, Army Military Medical University were enrolled in the study. Finally, 2,496 H&E-stained digital images were obtained after being labeled by senior pathologists, and each image size was 959 × 461 pixels. The image set was randomly divided as follows: a training set comprising 1,497 images was designated as the training data set for the deep learning model; a validation set comprising 499 images was used to assess the performance of PD-L1 state prediction and determine the optimal structure for the deep learning model; and a test set comprising 500 images was employed to evaluate the deep learning model’s segmentation and TPS prediction capabilities compared to the TPS as assessed by the pathologist.

### 2.2 Slice staining, scanning, and dataset labeling

Formalin fixed paraffin-embedded (FFPE) tissue samples of squamous cell carcinoma with known PD-L1 positive expression were successively sliced as 4 μm thick by technicians for H&E staining and PD-L1 IHC detection. H&E staining was performed using a fully automatic dyeing workstation Autostainer XL CV5030 (LEICA, Wetzlar, Germany). PD-L1 IHC staining was performed on the Dako Autostainer Link 48 platform (Dako, Copenhagen, Denmark) using a companion diagnostic kit (Antibody clone No. 22C3). Both H&E- and PD-L1-stained slices were generated digitally on a fully automated digital slice scanning system PRECISE 500B (UNIC-TECH, Beijing, China). The same areas on the H&E-stained digital images were synchronously labeled by trained pathologists using Adobe Photoshop CS6 software (Version 13.0), referring to the positive and negative tumor areas of PD-L1 expression on the IHC digital images. The labeled images were further confirmed by two senior pathologists as gold-standard.

**Figure 1:**
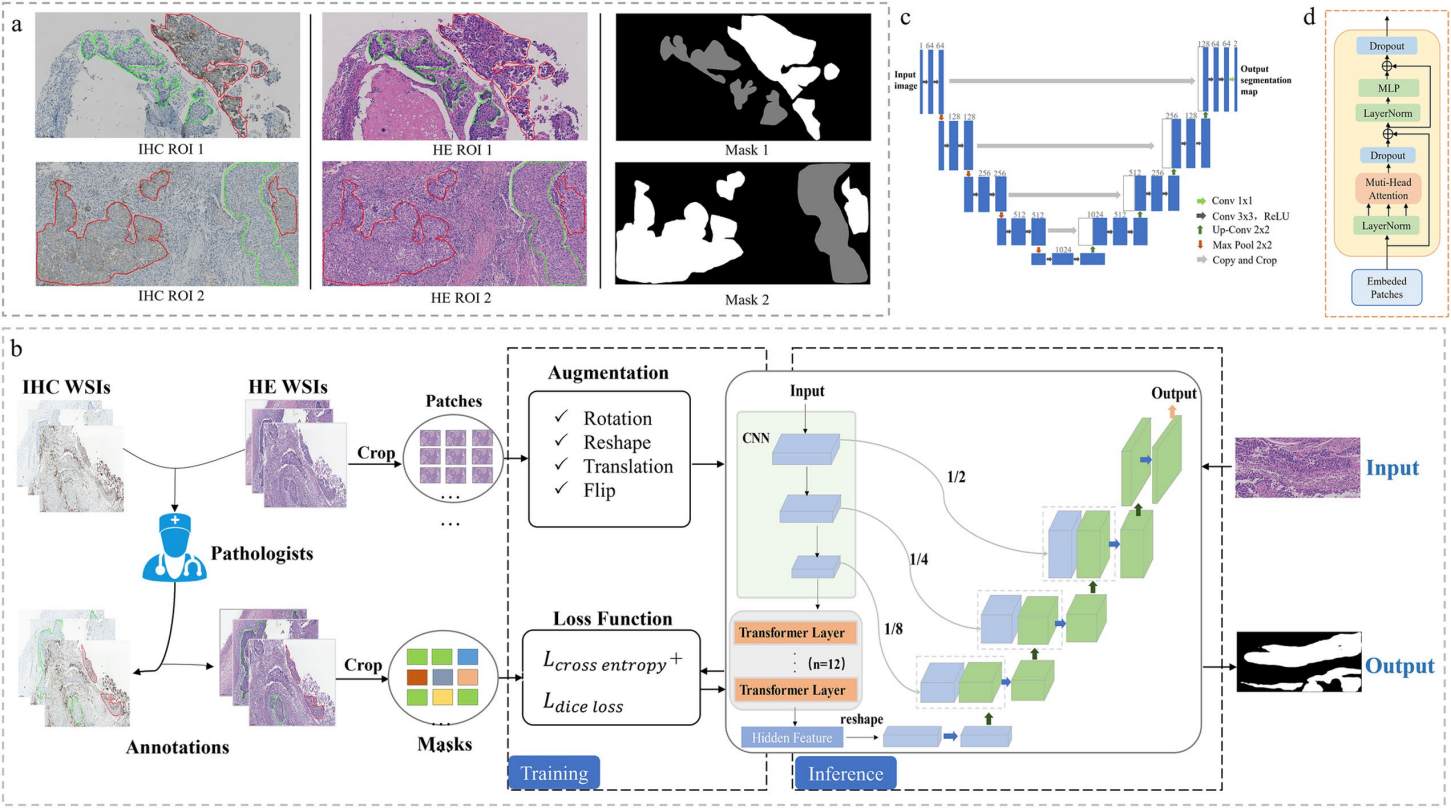
Annotated dataset generation and tumor segmentation framework construction. **(A)** Annotated pathological dataset of lung squamous carcinoma (the region of interest (ROI) labeled by pathologists based on IHC images, and synchronous generate ROI on H&E images and masks by the algorithm). Green line and the gray area: PD-L1 negative, red line and the white area: PD-L1 positive. **(B)** The entire framework of TransUnet. **(C)** Structure of Unet. **(D)** Structure of Transformer.

The region of interest (ROI) labeled by pathologists for IHC and H&E images and the mask generated by the algorithm are illustrated in Figure 1A. The area marked by the green line in the ROI map and the gray area of the mask generated by the algorithm are tumor regions with negative PD-L1 expression, whereas the area marked by the red line and the white area of the mask are tumor regions with positive PD-L1 expression.

### 2.3 Deep learning model development

#### 2.3.1 TransUnet framework

Focusing on the two problems of WSI resolution being too high for direct downsampling and patch-level classification lacking detailed representation, we proposed a more comprehensive solution for completing the recognition and segmentation of PD-L1 status in pathological images. The comprehensive detection framework is illustrated in Figure 1B, where black indicates background, normal tissue, white indicates PD-L1-positive areas of the tumor and gray indicates PD-L1-negative areas of the tumor.

(1) During the data collection process, H&E and PD-L1 IHC staining were performed on successive sections of FFPE tumor tissues. WSI images were generated using an automatic section scanner. According to the PD-L1 IHC digital images, the pathologists synchronically masked the positive and negative PD-L1 expression regions in the H&E images according to IHC images to obtain labeled WSI images. The data were further processed to satisfy the model. First, we used the flood fill algorithm to fill the ROI region in the WSI with the same pixel and then generated the mask label required by the training model. Using the sliding window algorithm, the original WSI images were divided into patches with no overlapping regions, and the size of the PD-L1 expression region in each patch was evaluated. Patches with no and few targets were discarded to avoid introducing many irrelevant targets, ensuring that the remaining patches had both background and target to better guide model learning.
(2) During model training, the segmented patch was first enhanced with data such as translation, rotation, and flip in random proportions. Subsequently, the depth segmentation network model TransUnet was trained based on the enhanced patch, and the combined loss of dice (Sudre et al., 2017) and Cross Entropy (CE) was adopted as the loss function. The former was responsible for measuring the overlap differences between the prediction results and label samples, while the latter calculated the pixel-level differences between the prediction results and label values. Combining the two losses can correctly guide model training and improve the segmentation model’s convergence speed. The model output was a pixel-by-pixel classification segmentation map, and more detailed WSI segmentation results were obtained.
(3) During model inference, the model input was the original patch images segmented after H&E staining, and the output was the segmentation results combined with the original patches. The segmentation results included two types of targets: the PD-L1 positive region and the PD-L1 negative region. The TPS of a patch can be quantitatively analyzed by calculating the pixel ratio of the positive area to the overall tumor area, and the TPS score of the entire WSI image can be obtained by combining the results of each patch.

We designed the framework by adopting the TransUnet network model with an encoder-decoder structure to predict and segment the negative/positive expression region of PD-L1, which was built based on Unet (Zunair and Hamza, 2021). The encoder included the following: The backbone network adopted the ResNet-V2 (He et al., 2016a,b) model. Compared with the traditional ResNet (He et al., 2016a,b) model, a pre-activation design was added to improve the performance of the residual module. Furthermore, transformer module was introduced after ResNet to enhance the modeling of context information and enrich the expression of long-range dependent information. The decoder included the following: The low-resolution feature map of the upper layer was continuously amplified by four layers of upsampling because the sampling process produced the problem of spatial structure information loss, and the shallow detailed spatial texture representation was transmitted to the upper layer with the same resolution through the shallow three skip connections. The scaling ratios of the three-hop connection were 1/2, 1/4, and 1/8. After the skip connection, the number of channels was doubled. In this case, 3 × 3 convolution refined information and compressed the number of channels to reduce the complexity of the model and improve the convergence speed. Multiscale features were captured by multiple sampling between the encoder and decoder. Then the complementary characteristics of deep and shallow, strong and weak were also taken into account. After fusion and refining these features, outputs feature maps with rich discriminant information.

#### 2.3.2 Unet structure

Unet (Zunair and Hamza, 2021) is a symmetric segmentation network that fuzes shallow structural features and deep semantic features with skip connections and effectively uses different features for segmentation prediction. The network mainly includes an encoder, decoder, and hop connection. As demonstrated in Figure 1C, the network consists of four downsampling and four upsampling modules. The downsampling module extracted sophisticated image features, reduced image resolution, and expanded information channels. The upsampling module recovered feature information and simultaneously fused the features of the previous layer to supplement the details lost in the compression process (Zhou et al., 2020).

#### 2.3.3 Transformer structure

Transformer (Vaswani et al., 2017) was first introduced in 2017 for natural language processing and have been widely used in computer vision in recent years. It adopts the structure of an encoder and a decoder, and the module structure is indicated in Figure 1D. The transformer mainly comprises position coding, layer regularization, a multihead attention mechanism, and a feedforward neural network. This uniquely designed transformer architecture can effectively capture long-distance dependencies and extract global features of images. Convolutional neural networks have significant advantages in extracting underlying features and obtaining local information. Therefore, a reasonable combination of convolutional operation and transformer modules can effectively compensate for their respective defects and fully exploit their advantages (Huang et al., 2022; Huang et al., 2023).

### 2.4 Segmentation effect comparison test and evaluation parameters

We selected several deep segmentation models to participate in the construction of the PD-L1 expression detection framework, including Unet (Zunair and Hamza, 2021), AttUnet (Oktay et al., 2018), TransUnet (Chen et al., 2021), FCN (Shelhamer et al., 2017), DeepLabv3 (Chen et al., 2017), DeepLabV3+ (Chen et al., 2018), DenseASPP (Yang et al., 2018), SETR (Zheng et al., 2020), Segmenter (Strudel et al., 2021), OCRNet (Wang J. et al., 2019; Wang S. et al., 2019), Segformer (Xie et al., 2021), BiseNetV2 (Changqian et al., 2021), and DDRNet (Pan et al., 2023). The Unet series models employed an encoder-decoder structure and integrated the recovered feature map with additional abundant features from the front layer. This approach facilitated the edge structure’s refinement. The DeepLab series models were characterized by the introduction of atrous spatial pyramid pooling (ASPP), which can improve the representation of multiscale information. To assess the efficacy of each segmentation model and select an optimal model for the construction of the PD-L1 prediction framework. We calculated the dice similarity coefficient (DSC), intersection overunion (IoU), accuracy pixel (AP), and Hausdorff distance (HD) to evaluate each model comprehensively. The calculation principle of each parameter index is as follows:


(1) $$\mathrm{Dice}=\frac{2|pred_{\mathrm{mask}}\cap \mathrm{mask}|}{\left|pred_{\mathrm{mask}}|+|\mathrm{mask}\right|}$$

(2) $$IoU=\frac{|pred_{\mathrm{mask}}\cap \mathrm{mask}|}{\left|pred_{\mathrm{mask}}\cup \mathrm{mask}\right|}$$

In Formulas 1 and 2, $pred_{\mathrm{mask}}$ represents the segmentation result predicted by the model, and the mask represents the true expression state of PD-L1 in the sample.


(3) $$\mathrm{Pixel}\;\mathrm{Accuracy}\;\left(PA\right)=\frac{\sum_{k=0}^{n}P_{k}}{\sum_{i=0}^{m}\sum_{j=0}^{n}P_{ij}}$$

In Formula 3, $P_{k}$ represents the pixels predicted correctly by the model and $P_{ij}$ represents all pixels in the output image of the model.


(4) $$\begin{array}{l} d_{H}\left(X,Y\right)=\max\left\{d_{XY},d_{YX}\right\}=\\ \max\left\{\begin{array}{l} \max_{x\in X}\min_{y\in Y}d\left(x,y\right),\\ \max_{y\in Y}\min_{x\in X}d\left(x,y\right) \end{array}\right\} \end{array}$$

In Formula 4, $d_{XY}\;\mathrm{and}\;d_{YX}\text{,}$ respectively, represents the distance between *x* set external and *y* set inside, and the distance between *y* set external and *x* set inside.

### 2.5 TPS consistency analysis

Five pathologists from Daping Hospital blindly evaluated TPS using IHC slides, and AI predicted TPS based on H&E slides in the test set. The root mean square error (RMSE) between the TPS evaluated by the five pathologists and the TPS predicted by AI with the gold standard of labeling labels was calculated. In the consistency analysis, intra-group correlation coefficients (ICC) were calculated between TPS predicted by AI and the gold standard and TPS evaluated by five pathologists (3,1). A total of 453 effective samples remained after preprocessing the digital slides and excluding 39 images with PD-L1 positive and negative proportions below 5% and 14 samples without sufficient tumor cells.

## 3 Results

### 3.1 Model performance and comparison test

The performance of each model on the test set is presented in Table 1. DeepLabv3+ (ResNet101) achieves poor segmentation accuracy because it uses single upsampling to integrate low-level details into high-level information, resulting in insufficient use of details. However, the Unet network continually combines bottom feature maps of varying resolutions with top layers to maximize the utilization of local details, thereby improving prediction accuracy. FCN (ResNet101), Attention Unet, and DeepLabv3 improve the effective feature extractor, introduce an attention mechanism to refine features, and expand the global receptive field, respectively, to further improve the segmentation effect of the network while retaining detailed information. Nevertheless, a common disadvantage of these models is that they fail to enhance the significance of global features in pathological image segmentation. To achieve the best segmentation effect, the TransUnet model employed in this study not only implemented the effective fusion of multiscale features but also enhanced the significance of global discriminant features in prediction. The DSC coefficient of overlap between PD-L1 expression regions predicted by the TransUnet model and real labels was 80%, the intersection ratio IoU was 72%, the edge accuracy was 89, and the average pixel accuracy was 88%. Compared with other models, TransUnet has apparent advantages regarding object recognition integrity and structural similarity (Table 1).

**Table 1:** Comparative experimental results of the different models.

Model	DSC/%	IoU/%	HD95	mPA/%	Weights/Mb	Parameters/10^6^
TransUnet	80.0	72.0	89	88.0	403	105
Unet	77.3	68.4	101	86.3	131	34
Attention Unet	78.7	69.6	101	78.7	133	35
DenseASPP	77.3	68.6	100	86.6	136	35
FCN(ResNet101)	78.3	69.2	99	86.8	198	52
DeepLabv3(MobileNetV3)	78.9	70.1	99	87.2	42	11
DeepLabv3 + (ResNet101)	75.0	65.9	104	85.2	224	58
DeepLabv3 + (HRNet)	77.5	68.7	100	86.3	274	71
SETR	76.1	53.2	143	64.9	1,177	309
Segmenter	75.2	52.5	205	66.9	327	86
OCRNet	74.0	48.5	144	64.6	51	13
Segformer	74.1	49.9	214	62.0	14	4
BiseNetV2	78.5	54.8	207	66.9	20	5
DDRNet	73.8	46.9	216	59.7	77	20

**Figure 2:**
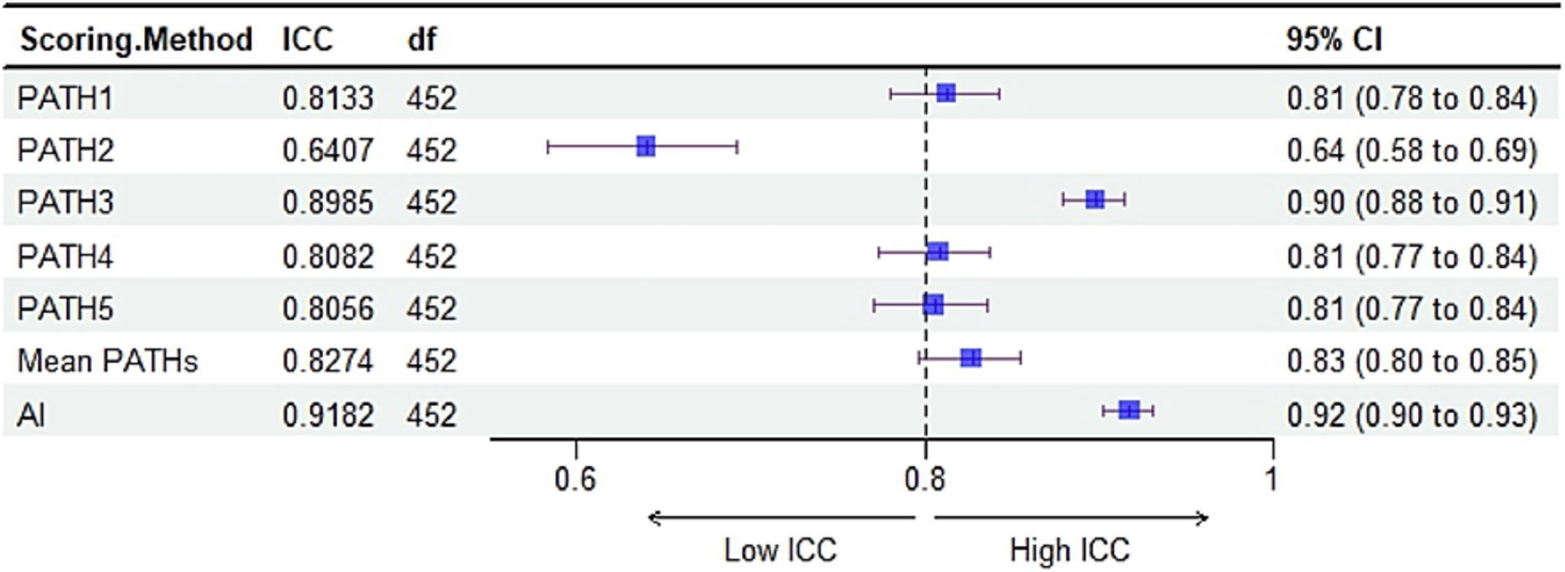
Pixel accuracy of positive/negative tumor segmentation category.

Figure 2 displays the pixel accuracy of the PD-L1 positive and negative regions for each model. The pixel recognition accuracy of TransUnet for negative and positive expression regions was 85 and 75%, respectively, and its accuracy was better than other models in both single and average categories. Accordingly, the expression status of PD-L1 can be identified more accurately.

**Figure 3:**
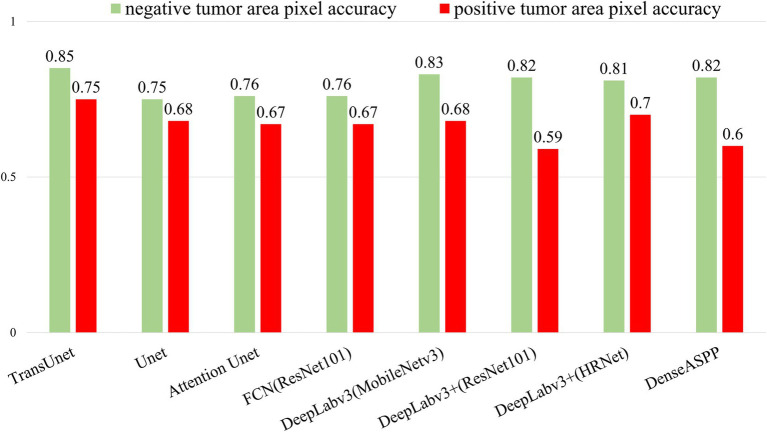
The comparative experiment of model segmentation effect. Green area: PD- L1 negative, Red area: PD-L1 positive.

**Figure 4:**
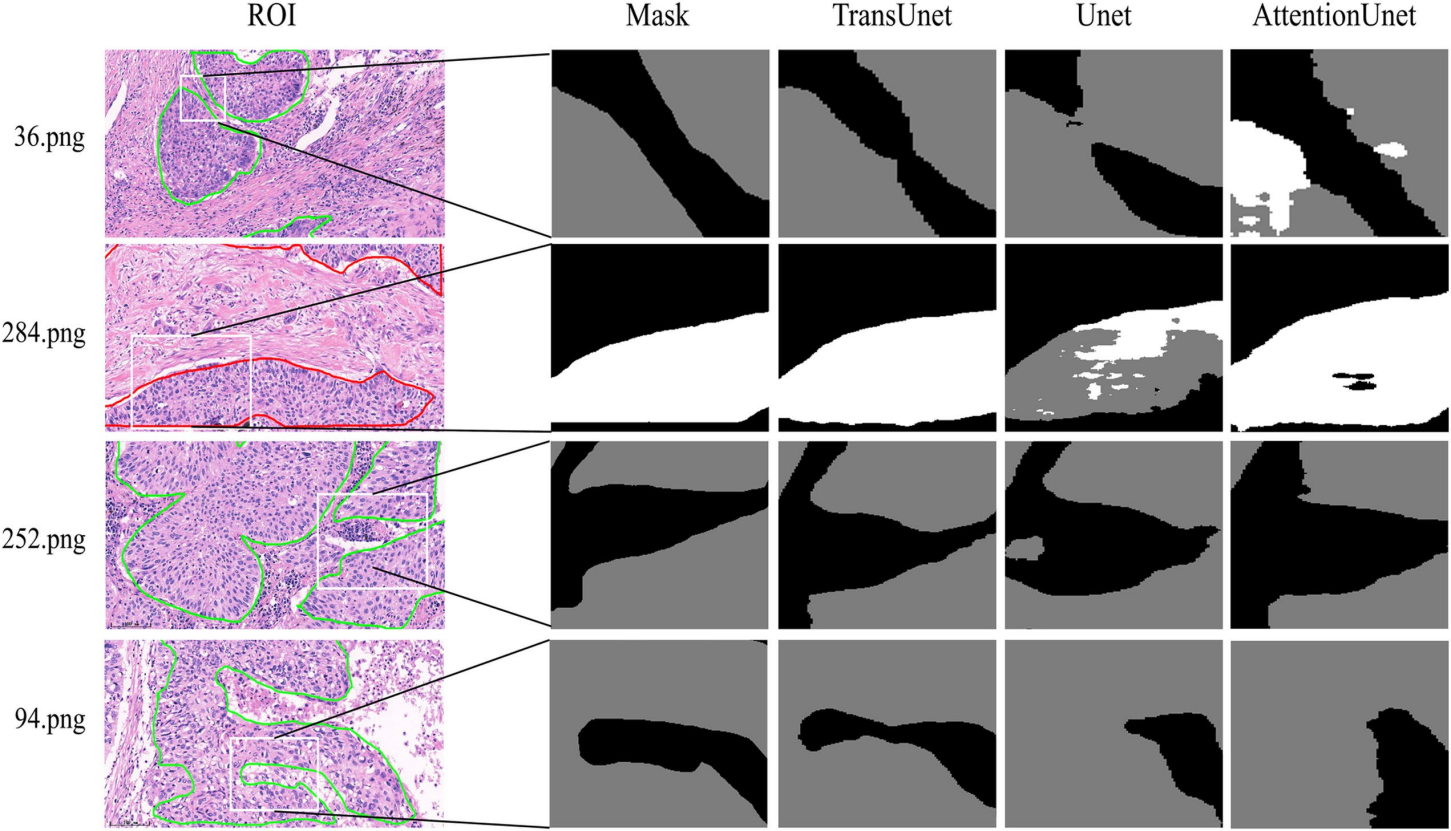
The comparative experiment of the segmentation details of three Unet models. Gray area: PD-L1 negative, White area: PD-L1 positive.

To visually compare the prediction accuracies of various models, we opted to visualize the model’s segmentation effect. The model’s prediction results are displayed in gray for PD-L1 negative regions and white for PD-L1 positive regions. The ROI represented the initial mark of the doctor, and a mask was generated by the algorithm to identify the ROI. The inaccurate segmentation of a large target area resulted from a detailed information deficiency in the prediction layer of DeepLabv3+ (ResNet101). The Attention Unet and FCN (ResNet101) greatly emphasized the extraction of detailed features, which weakened the ability to locate lesions and compromised the integrity of the segmentation target. DenseASPP and DeepLabv3+ (HRNet) exhibit improved segmentation accuracy compared with other models with less difference between the target and background (Figure 3). TransUnet emphasized not only global information but also detailed information acquisition, resulting in accurate segmentation results and more detailed segmentation of PD-L1 expression regions. Regarding the extent of segmentation detail reduction, TransUnet exhibited a distinct advantage over the other two Unet-based models (Figure 4).

### 3.2 TPS comparison test

The accuracy of TPS quantitatively predicted by AI using H&E-stained slides was compared with the TPS assessed by five senior pathologists. Five pathologists performed TPS evaluation blindly on IHC slides of 453 samples in the test set and recorded the results. Additionally, the TPS predicted quantitatively by AI was approximated by the ratio of the PD-L1 positive area to the total tumor area in the TransUnet inference segmentation results.

**Figure 5:**
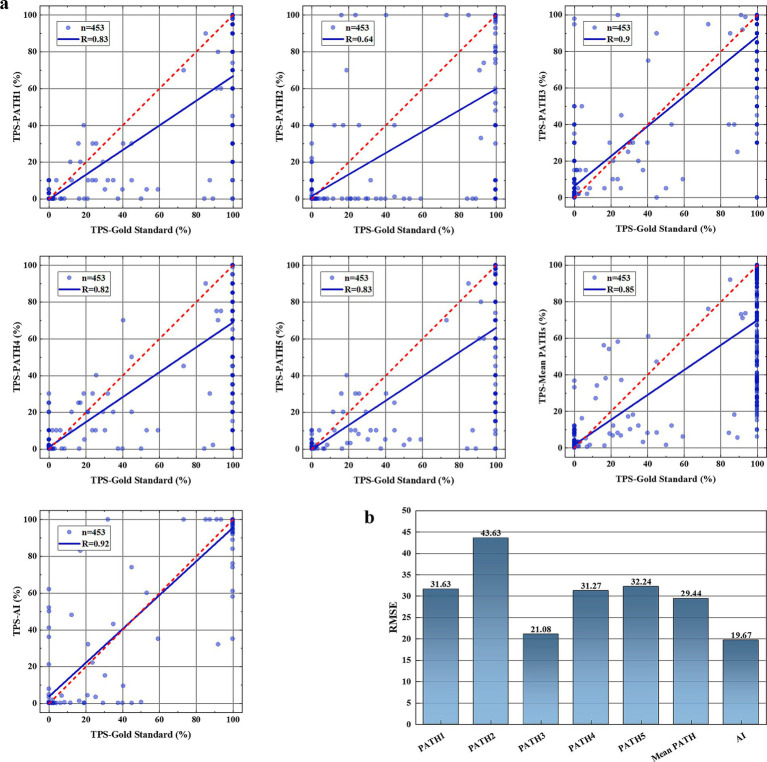
TPS comparison test results. **(A)** The consistency of TPS assessed by five pathologists (TPS-PATH N, N = 1–5), the mean TPS of five pathologists (TPS-mean PATHs), and AI-TPS with the gold standard TPS. **(B)** Root mean square error (RMSE) between TPS-PATH N (N = 1–5), TPS-mean PATHs, and TPS-AI with the gold standard TPS.

Five pathologists evaluated TPS (TPS-PATH N, N = 1–5); five pathologists evaluated mean TPS (TPS-mean PATHs); and the TPS evaluated by AI (TPS-AI) was compared to the gold standard to assess the diagnostic accuracy of pathologists and AI in TPS. The results showed that TPS-AI had the best agreement with the gold standard (TPS-AI vs. gold standard: R = 0.92), followed by TPS-PATH3 (TPS-PATH3 vs. gold standard: R = 0.9), and TPS-mean PATHs (TPS-mean PATHs vs. gold standard: R = 0.85) (Figure 5A). Where black indicates background, normal tissue. White indicates PD-L1-positive areas of the tumor. Gray indicates PD-L1-negative areas of the tumor.

Additionally, we calculated the RMSE between TPS-PATH N (N = 1–5) and the gold standard, between TPS-mean PATHs and the gold standard, and between TPS-AI and the gold standard to reflect the accuracy of TPS evaluated by TransUnet through H&E slides. As presented in Figure 5B, AI had the smallest RMSE (RMSE = 19.67) with the gold standard, which was lower than the best-performing pathologist (PATH3, RMSE = 21.08) and the TPS-mean PATHs (RMSE = 29.44).

### 3.3 Consistency analysis

**Figure 6:**
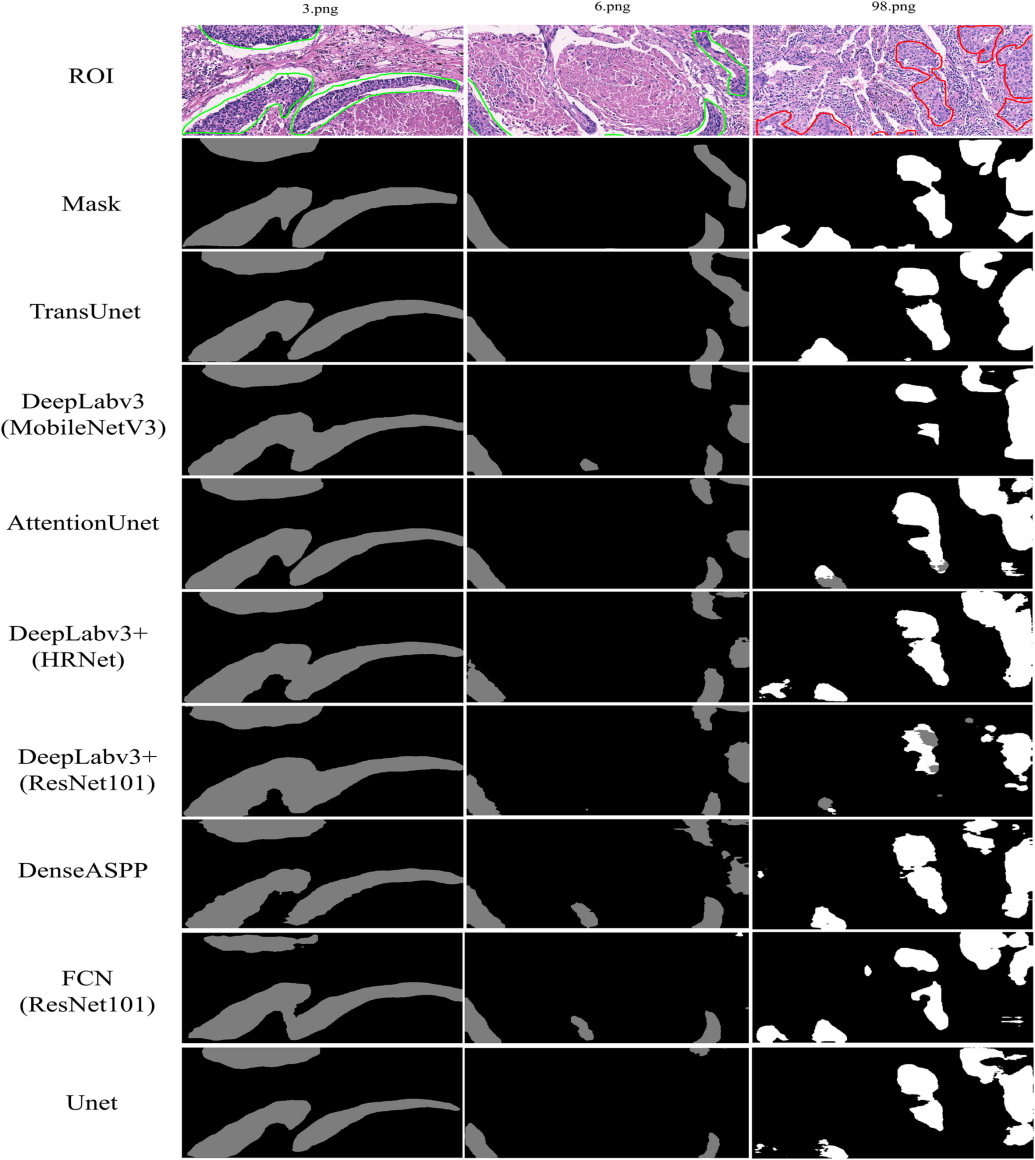
Intra-group correlation coefficient (ICC) between TPS-PATH N (N = 1–5), TPS-mean PATHs and TPS-AI, respectively, with the gold standard.

For the 453 samples in the test set, we calculated the ICC (3,1) between TPS-PATH N (N = 1–5), TPS-mean PATHs, and TPS-AI, respectively, with the gold standard. The results showed that AI had the best consistency with the gold standard, ICC (3,1) value was 0.92 (95% CI: 0.90–0.93) than PATH N or mean PATHs (Figure 6). Where black indicates background, normal tissue. White indicates PD-L1-positive areas of the tumor. Gray indicates PD-L1-negative areas of the tumor.

### 3.4 Generalization experiment

To verify the generalizability and reliability of the TransUnet model, we performed experiments on the laryngeal tumor pathology image dataset. TransUnet also demonstrates significant advantages in object recognition completeness and structural similarity (Table 2).

**Table 2:** Generalization experimental results of the different models.

Model	DSC/%	IoU/%	HD95	mPA/%	Weights/Mb	Parameters/10^6^
TransUnet	80.5	67.8	238	75.0	403	105
SETR	77.9	64.8	89	72.2	1,177	309
Segmenter	75.2	60.5	265	69.3	327	86
OCRNet	75.3	60.5	104	68.8	51	13
Segformer	73.8	58.8	254	67.3	14	4
BiseNetV2	77.6	64.5	222	71.3	20	5
DDRNet	76.9	63.3	240	70.4	77	20

## 4 Discussion

Under high magnification, the TPS evaluation of PD-L1 IHC-stained slides in lung squamous cell carcinoma is based on the accurate interpretation of tumor cells with positive cell membrane expression. However, the interpretation is hindered by necrotic and immune cells (e.g., lymphocytes and macrophages) that express PD-L1. Furthermore, determining the proportion of tumor cells exhibiting positive expression under low magnification is a challenging task, and pathologists’ diagnostic efficacy could be significantly compromised by frequent magnification changes on the microscope (Coudray et al., 2018; Fraz et al., 2020). Therefore, accurately identifying the TPS of PD-L1 expression at global and detailed levels using deep learning models is of significant clinical application value. Results of this research demonstrated that when segmenting PD-L1 status in pathological images, the convolutional neural network-based segmentation model emphasizes the overall integrity of the target instead of accurate learning of its details; thus, the structural similarity of the segmentation results has disadvantages. Particularly, the accuracy of segmentation was reduced when differences between the object and the background were minimal. By combining the benefits of Unet and Transformer technology, the TransUnet model is capable of not only accurately identifying regions of PD-L1 expression that are positive or negative (with the highest pixel recognition accuracy) but also presenting segmentation details in the segmentation results to ensure the target’s structural similarity is maintained.

Considering the effects on image segmentation and recognition and the degree of restoration, some models were inaccurate in the segmentation of large areas because of the lack of attention to details. Nevertheless, certain models have laid an excessive emphasis on detailed feature extraction, resulting in compromised segmentation targets due to weakened lesion localization abilities. Compared with these models, TransUnet segmented and identified PD-L1 expression regions with more accuracy. Compared to the Unet and AttentionUnet models, segmentation and recognition at the edge of the target region are more accurate, and the reduction with mask labels is significant.

Nevertheless, the clinical application of IHC staining is challenging due to its complicated process, long duration, lack of standardization of interpretation standards, and most importantly, its heavy reliance on subjective evaluation by experienced pathologists. Accurate prediction of PD-L1 status using deep learning models to analyze tissue and cell morphology in H&E-stained sections will undoubtedly facilitate the clinical application of PD-L1 expression scores. Although the TransUnet model applied to PD-L1 TPS prediction in this sdudy still needs to be trained with large number of cases, and confirmed in large-scale independent cohort studies. The current results showed that the model integrating the advantages of Unet and Transformer technology can potentially predict PD-L1 expression using H&E-stained digital slides. More importantly, compared with the subjective assessment of pathologists, the prediction results of PD-L1 TPS status using the TransUnet model are always objective and repeatable. Furthermore, this model is not only suitable for the prediction of PD-L1 in lung squamous cell carcinoma, but can also be applied to other cancers and different biomarkers based on deep learning, thus providing a new path for the description of histopathological characteristics of biomarkers and clinical treatment target screening.

This study has certain limitations, and we hope to solve these problems during continuous training and clinical cohort studies. First, due to the insufficient number of images in the training group, the TransUnet model’s ability to accurately predict PD-L1 expression in lung squamous cell carcinoma remains inadequately representative. Second, deep learning and testing of PD-L1 expression in lung adenocarcinoma are lacking. Compared with lung squamous cell carcinoma, lung adenocarcinoma is more complex and heterogeneous, which undoubtedly poses particular challenges for the deep learning and prediction of this model. Nevertheless, the complexity and heterogeneity of lung adenocarcinoma tissue structure are challenging and subject to interpretation differences for pathologists under light microscopy. Therefore, a head-to-head comparison of PD-L1 TPS between this model and the pathologists in the real working environment is of great clinical significance, and it is also helpful for correcting the inconsistent results of the pathologist.

## 5 Conclusion

We proposed TransUnet, a framework for quantitative PD-L1 TPS prediction using H&E-stained digital images. The backbone of the framework is the TransUnet semantic segmentation model. TransUnet enhanced the modeling of global context information in the encoder stage and combined the representation of local features using a CNN. In the decoder stage, the feature map was amplified by multiple upsampling, and the features of the shallow and high layers were fuzed to acquire more discriminative features. The DSC coefficient and IoU of this model for PD-L1 segmentation prediction based on H&E images of lung squamous cell carcinoma were 80 and 72%, respectively, which ensured the integrity of the segmentation target and improved its structural similarity. Furthermore, when comparing the deep learning network’s quantitative prediction of TPS to that of five pathologists assessment, it exhibited a greater degree of concurrence with the gold standard [ICC (3,1) = 0.92, 95% CI: 0.90–0.93] and a reduced error (RMSE = 19.67). AI not only has good accuracy in predicting TPS but also can eliminate the evaluation errors caused by subjective differences between different pathologists. In conclusion, the deep learning network proposed in this study can effectively assist pathologists in completing PD-L1 TPS prediction using H&E-stained digital images.

## Data Availability

The raw data supporting the conclusions of this article will be made available by the authors, without undue reservation.
